# Supplementary material for: Metagenomic sequencing reveals a lack of virus exchange between native and invasive freshwater fish across the Murray–Darling Basin, Australia
Source: Virus Evol. 2021 Apr 13;7(1):veab034. doi: 10.1093/ve/veab034 (PMC8121191; doi:10.1093/ve/veab034)
Supplement: veab034_Supplementary_Data [file veab034_supplementary_data.zip › SITable1.pdf]

Host	Virus name	Virus family	Gene/ORF	Length (nt)	Closest relative (NCBI/Genbank)	Amino acid similarity (%)
Western carp-gudgeon (*Hypseleotris spp.)*	*Western carp-gudgeon arenavirus*	*Arenaviridae*	L protein (RdRp)	832	*Wenling frogfish arenavirus 1* (YP_009551555)	36.9
Eastern mosquitofish (*Gambusia holbrooki*)	*Eastern mosquitofish arenavirus*	*Arenaviridae*	L protein (RdRp)<br><br>Nucleoprotein	5991<br><br>1884	*Wenling frogfish arenavirus 1* (YP_009551555)<br><br>*Wenling frogfish arenavirus 1* (YP_009551555)	84.5<br><br>78.7
Spangled perch (*Leiopotherapon unicolor*)	*Spangled perch bastrovirus*	*Astroviridae*	Non-structural polyprotein	1294	*Bastrovirus-like virus Vietnam Bat* (YP_009333174.1)	81.3
Eastern mosquitofish (*Gambusia holbrooki*)	*Eastern mosquitofish bastrovirus*	*Astroviridae*	Non-structural polyprotein<br><br>Structural polyprotein	2463<br><br>563	*Bastrovirus Brazil/sewage* (ASM79505)<br><br>*Bastrovirus Brazil/sewage* (ASM79506)	61.7<br><br>75.4
Murray-Darling rainbowfish (*Melanotaenia fluviatilis)*	*Murray-Darling rainbowfish astrovirus*	*Astroviridae*	RdRp	1989	*Wuhan astro-like virus* (AVM87125)	40.3
Common carp (*Cyprinus carpio*)	*Murray-Darling carp cultervirus*	*Bornaviridae*	L protein (RdRp)<br><br>Glycoprotein	5190<br><br>1575	*Sharpbelly cultervirus* (AVM87541)<br><br>*Sharpbelly cultervirus* (AVM87539)	93.3<br><br>86.8

			Nucleoprotein	1095	*Sharpbelly cultervirus* (AVM87536)	92.9
Bony herring (*Nematalosa erebi*)	*Bony herring calicivirus*	*Caliciviridae*	Polyprotein	387	*Atlantic salmon calicivirus* (AHX24377)	80.3
Common carp (*Cyprinus carpio*)	*Murray-Darling carp letovirus*	*Coronaviridae*	RdRp	228	*Pacific salmon nidovirus* (QEG08237)	50.7
Unspecked hardyhead (*Craterocephalus fulvus*)	*Hardyhead chuvirus*	*Chuviridae*	L protein (RdRp)<br><br>Glycoprotein<br><br>Nucleoprotein	6363<br><br>1956<br><br>1566	*Guangdong red-banded snake chuvirus-like virus* (AVM87272)<br><br>*Wenling fish chu-like virus* (AVM87276)<br><br>*Herr Frank virus* (QHX39758)	44<br><br>41<br><br>34
Western carp-gudgeon (*Hypseleotris spp.)*	*Western carp-gudgeon flavivirus*	*Flaviviridae*	NS5	1866	*Cyclopterus lumpus virus* (ATQ64261)	36
Murray-Darling rainbowfish (*Melanotaenia fluviatilis*)	*Murray-Darling rainbowfish hantavirus*	*Hantaviridae*	RdRp	1116	*Bern perch virus* (QGM12349)	27.3
Common carp (*Cyprinus carpio*)	*Murray-Darling carp hepevirus*	*Hepeviridae*	Polyprotein (RdRp)	2682	*Cutthroat trout virus* (YP_004464929)	31.1
Eastern mosquitofish (*Gambusia holbrooki*)	*Eastern mosquitofish hepevirus*	*Hepeviridae*	Polyprotein (RdRp)	6456	*Wenling thamnaconus septentrionalis hepevirus* (AVM87557.1)	29
Western carp-gudgeon (*Hypseleotris spp.)*	*Western carp-gudgeon paramyxovirus*	*Paramyxoviridae*	RdRp	512	*Wenling tonguesole paramyxovirus* (AVM87378)	35.2

Australian smelt (*Retropinna semoni*)	*Australian smelt picornavirus*	*Picornaviridae*	RdRp	357	*Eel picornavirus* (YP_008531322)	54.2
Western carp-gudgeon (*Hypseleotris spp.)*	*Western carp-gudgeon poxvirus*	*Poxviridae*	DNA polymerase	213	*Salmon gill poxvirus* (YP_009162448.1)	61.4
			DNA-dependant RNA polymerase subunit rpo22	340	*Salmon gill poxvirus* (YP_009162433)	46.5
			DNA-dependant RNA polymerase subunit rpo19	226	*Salmon gill poxvirus* (YP_009162475)	40
			DNA-binding virion core protein I1L	643	*Salmon gill poxvirus* (YP_009162452)	28.1
			Myristylated protein A16L	247	*Salmon gill poxvirus* (YP_009162490.1)	32.9
			Hypothetical protein (SGPV079)	289	*Salmon gill poxvirus* (YP_009162451.1)	40.9
Common carp (*Cyprinus carpio*)	*Murray-Darling carp reovirus*	*Reoviridae*	RdRp	360	*Wenling scaldfish reovirus (AVM87459)*	40
Common carp (*Cyprinus carpio)*	*Murray-Darling carp rhabdovirus*	*Rhabdoviridae*	RdRp	592	*Beihai dimarhabdovirus 1* (AVM87284)	35.7
