# Supplementary material for: Metagenomic sequencing reveals a lack of virus exchange between native and invasive freshwater fish across the Murray–Darling Basin, Australia
Source: Virus Evol. 2021 Apr 13;7(1):veab034. doi: 10.1093/ve/veab034 (PMC8121191; doi:10.1093/ve/veab034)
Supplement: veab034_Supplementary_Data [file veab034_supplementary_data.zip › SITable2.pdf]

Library	Site	Fish	Replicates	RNA concentration (ng/ul)
L1	Lake Keepit	Common carp	5	183.56
L2	Lake Keepit	Bony herring	5	457.31
L3	Narrabri Creek	Common carp	7	709.61
L4	Narrabri Creek	Bony herring	4	537.85
L5	Narrabri Creek	Western carp-gudgeon	5	451.44
L6	Narrabri Creek	Australian smelt	5	153.54
L7	Narrabri Creek	Spangled perch	1	339.90
L8	Narrabri Creek	Goldfish	5	227.57
L9	Barwon River	Bony herring	3	504.82
L10	Barwon River	Western carp-gudgeon	4	378.86
L11	Barwon River	Common carp	8	259.12
L12	Bogan River	Common carp	8	427.19
L13	Bogan River	Bony herring	5	929.30
L14	Bogan River	Western carp-gudgeon	5	676.06
L15	Castlereagh River	Eastern mosquitofish	9	173.52
L16	Castlereagh River	Western carp-gudgeon	6	101.78
L17	Castlereagh River	Common carp	10	716.04
L18	Macquarie River	Common carp	10	202.85
L19	Macquarie River	Eastern mosquitofish	6	355.86
L20	Gwydir River	Bony herring	3	344.41
L21	Gwydir River	Common carp	5	309.72

L22	Lake Burrendong	Common carp	5	137.24
L23	Abercrombie River	Common carp	2	233.71
L24	Murray River (Nursery bend)	Murray-Darling rainbowfish	5	113.42
L25	Murray River (Nursery bend)	Common carp	4	167.15
L26	Edward River	Common carp	4	519.99
L27	Edward River	Murray-Darling rainbowfish	5	290.21
L28	Edward River	Australian smelt	2	130.65
L29	Edward River	Unspecked hardyhead	6	319.00
L30	Murray River (Wemen)	Murray-Darling rainbowfish	3	148.63
L31	Murray River (Wemen)	Flat-headed gudgeon	7	204.57
L32	Murray River (Wemen)	Australian smelt	5	166.38
L33	Murray River (Wemen)	Common carp	2	222.03
L34	Murray River (Coomealla)	Murray-Darling rainbowfish	4	152.49
L35	Murray River (Coomealla)	Flat-headed gudgeon	2	199.13
L36	Murray River (Coomealla)	Common carp	4	179.50
