# Supplementary figures and images for: Metagenomic sequencing reveals a lack of virus exchange between native and invasive freshwater fish across the Murray–Darling Basin, Australia
Source: Virus Evol. 2021 Apr 13;7(1):veab034. doi: 10.1093/ve/veab034 (PMC8121191; doi:10.1093/ve/veab034)

*Rhabdoviridae*

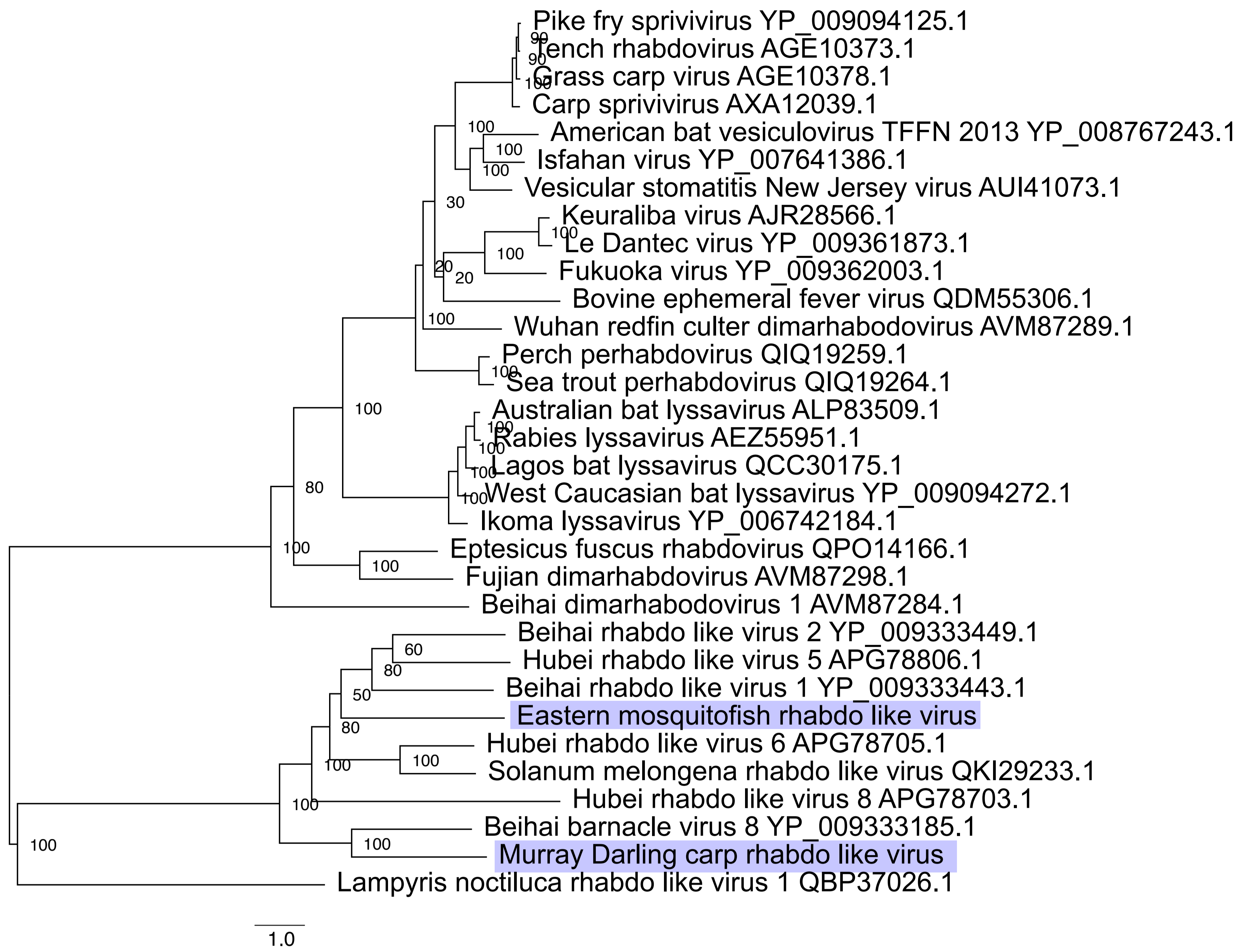

*Picornaviridae*

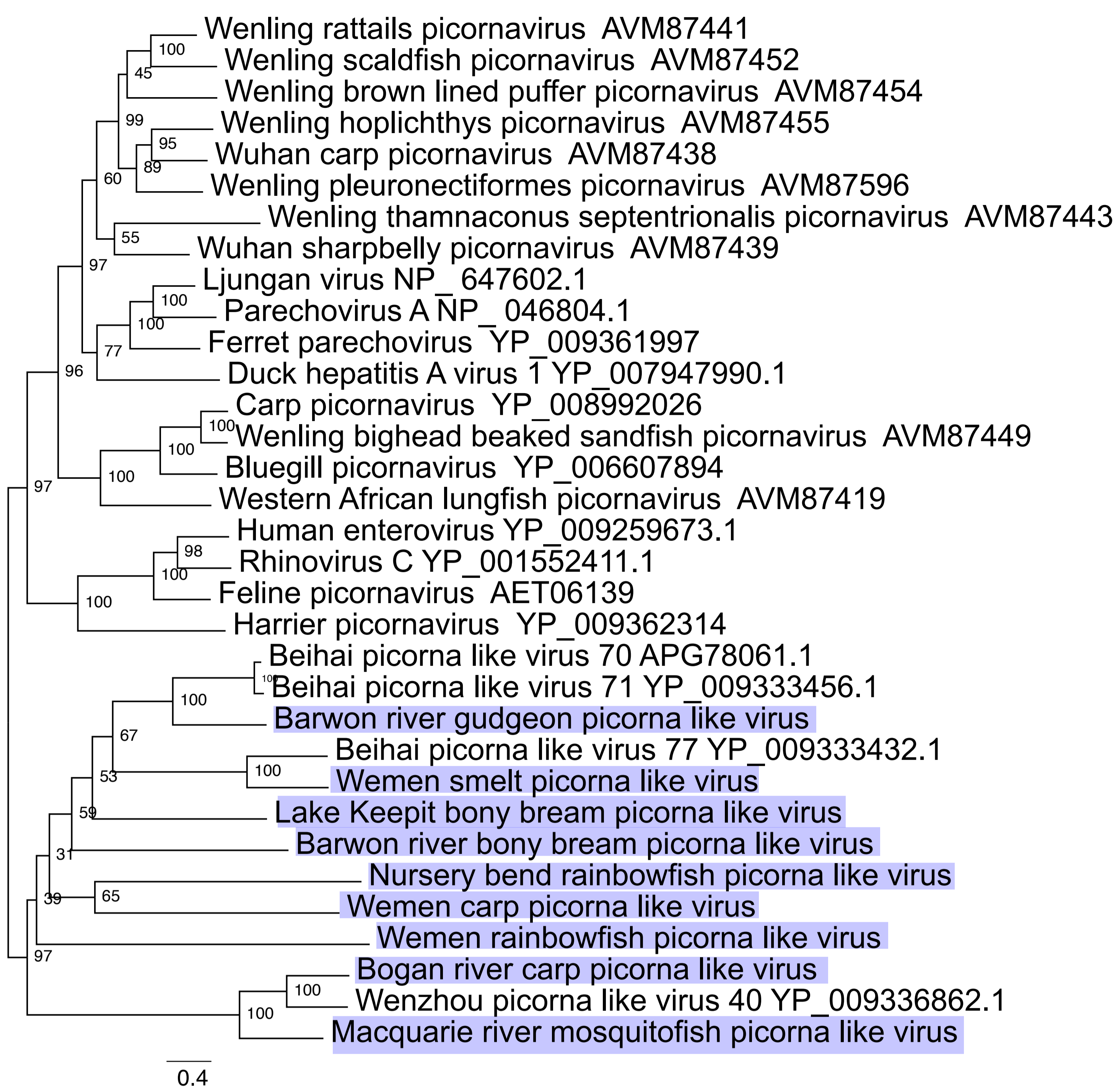

*Narnaviridae*

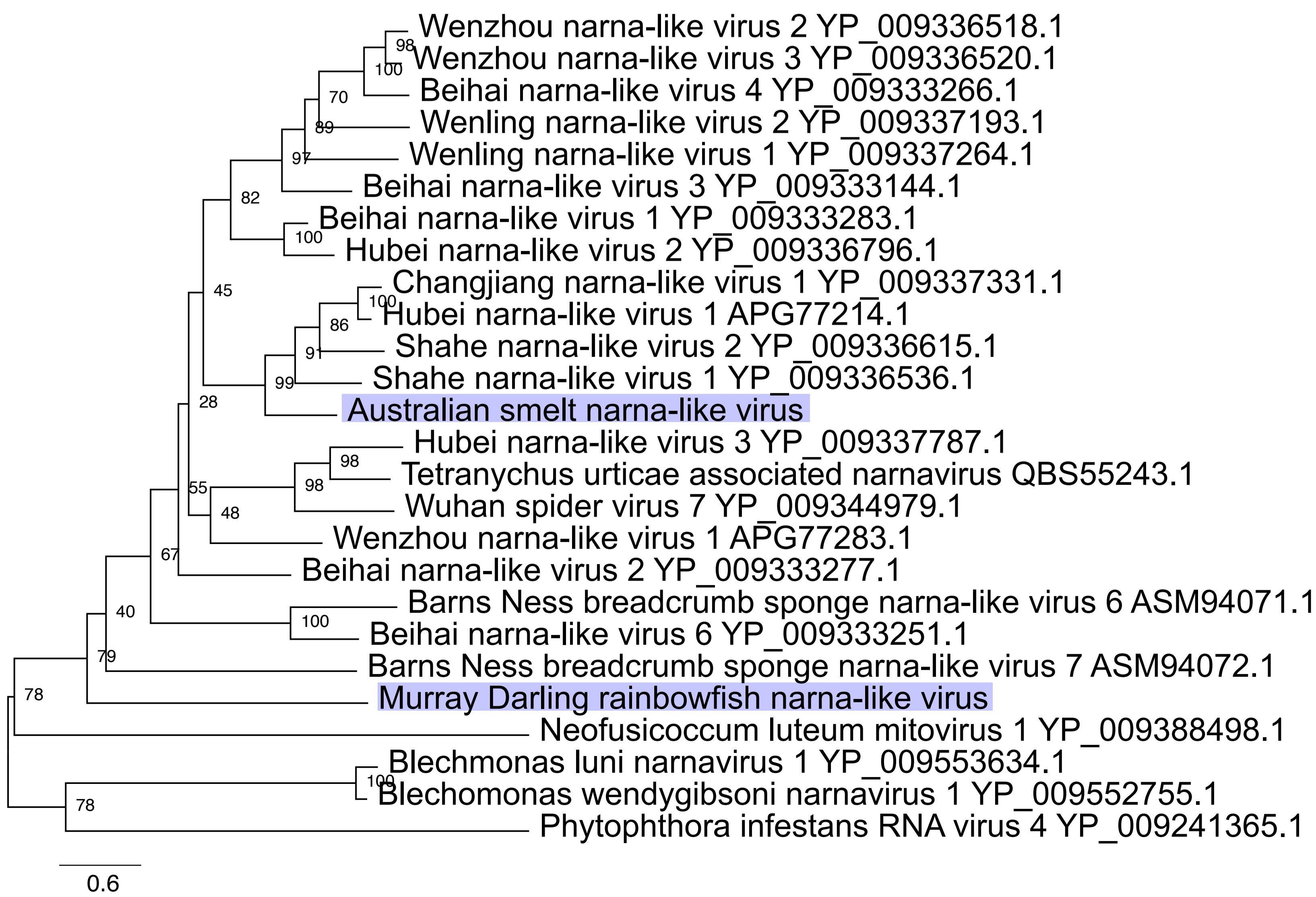

*Tombusviridae*

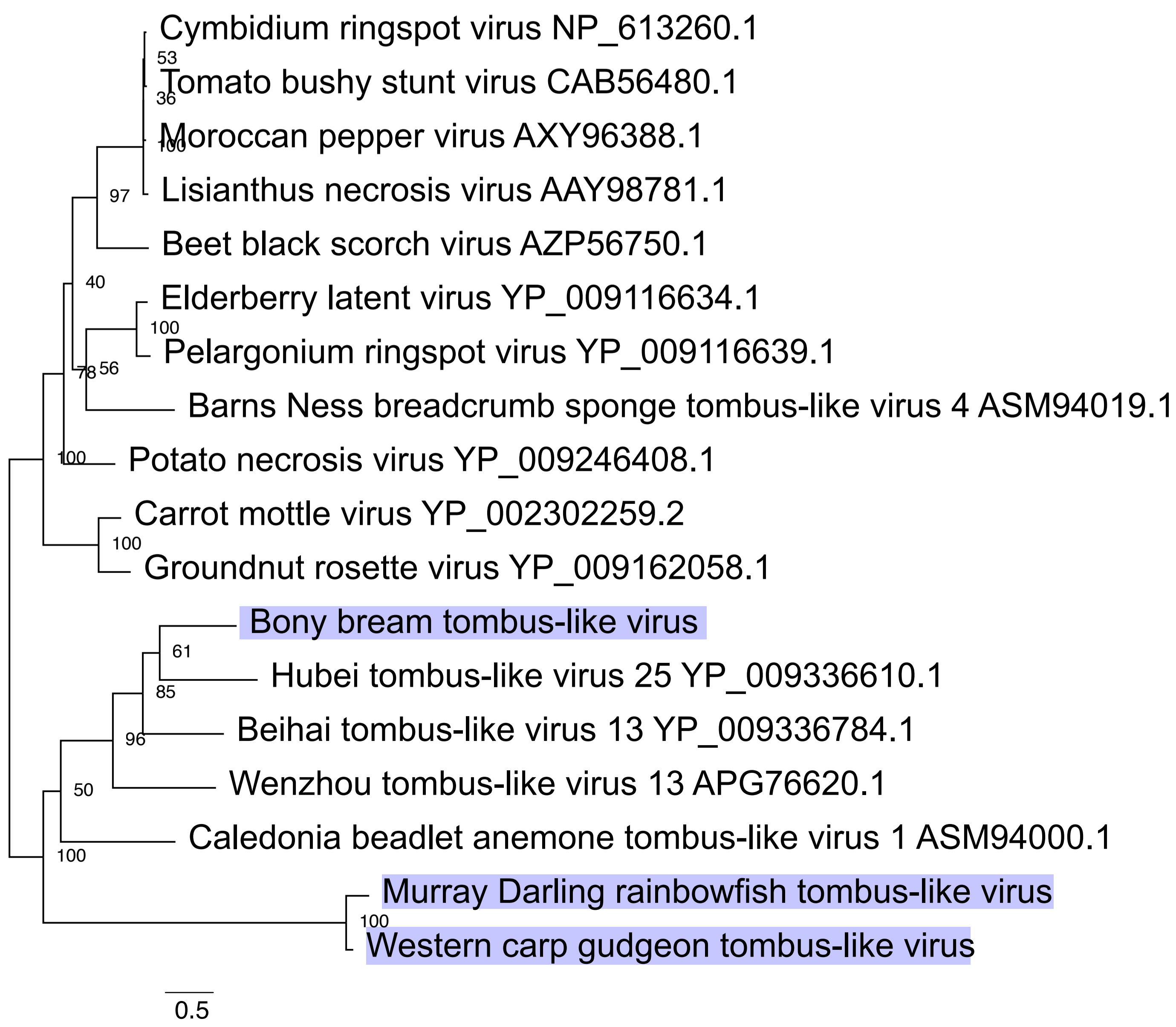

Supplement: veab034_Supplementary_Data [file veab034_supplementary_data.zip › SIFigure1.pdf]

*Nodaviridae*

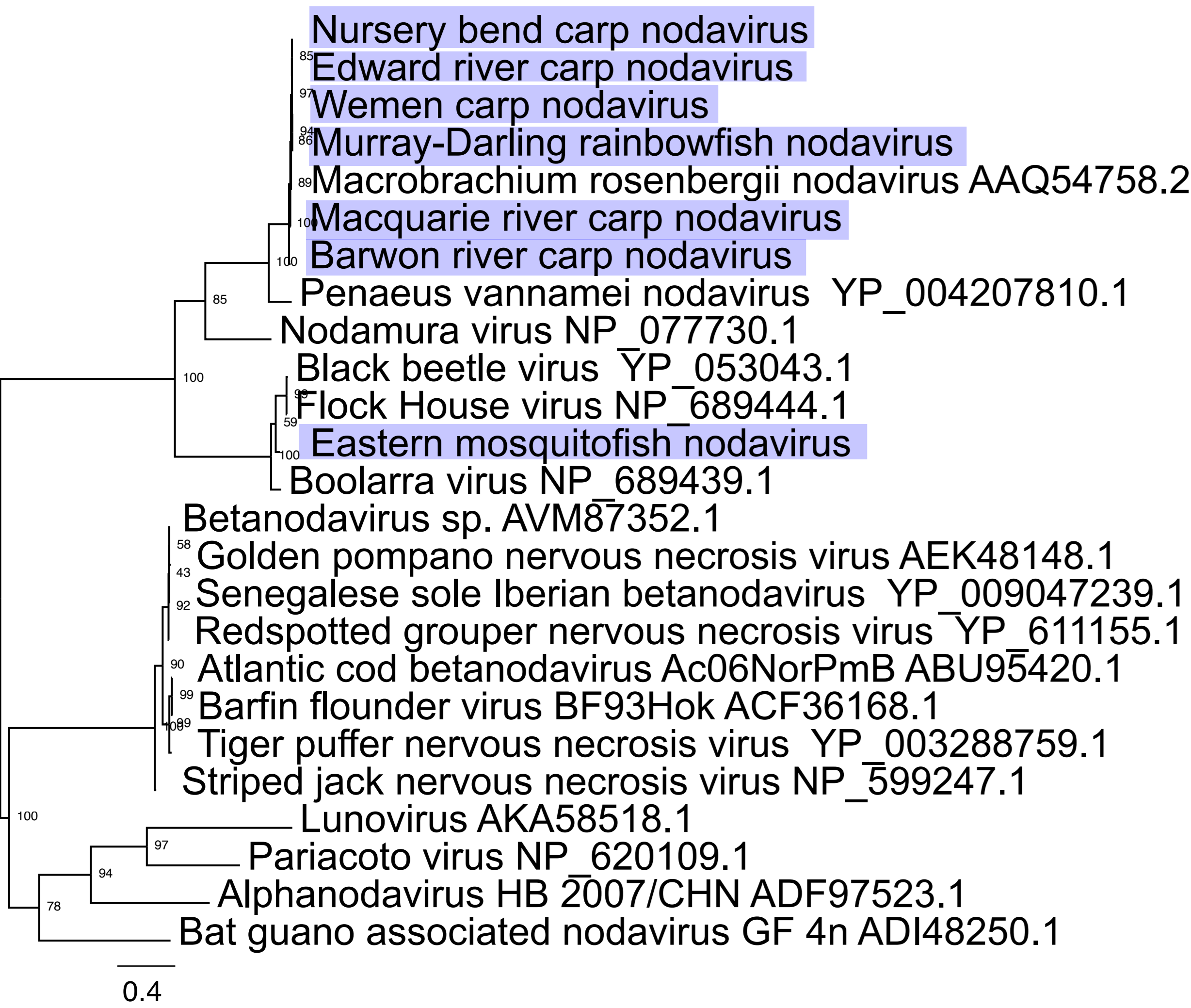

*Phenuiviridae*

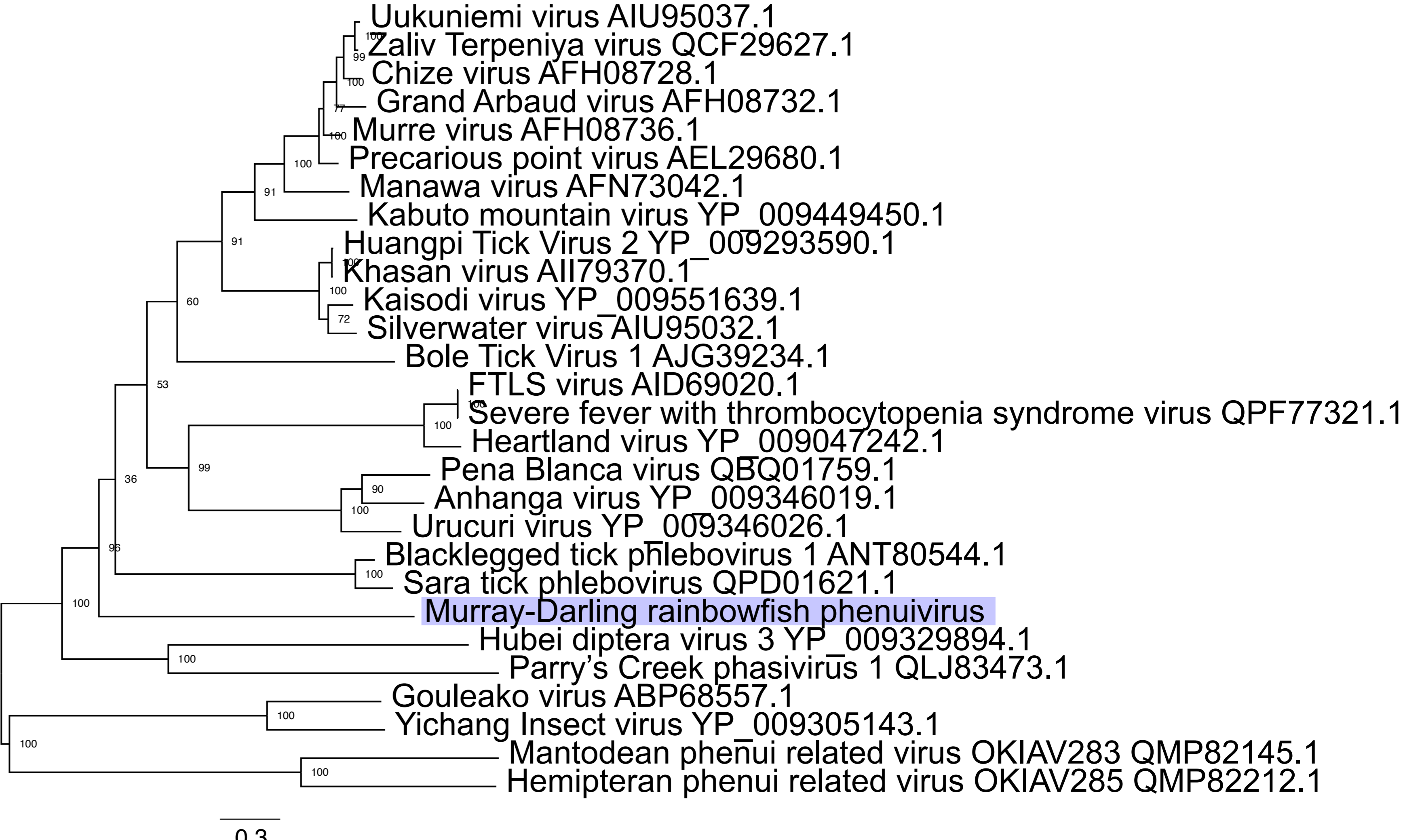

*Dicistroviridae*

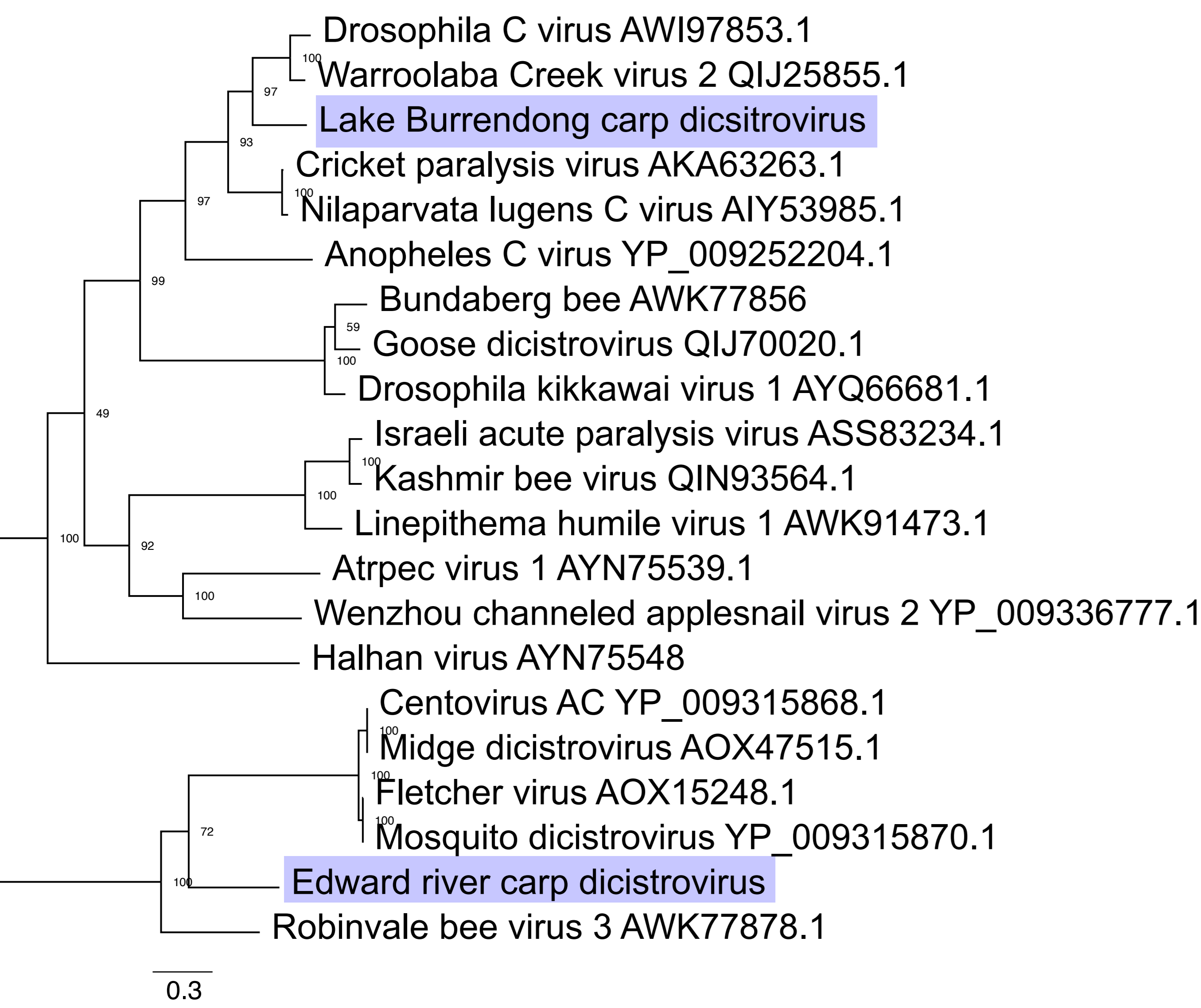

*Permutotetraviridae*

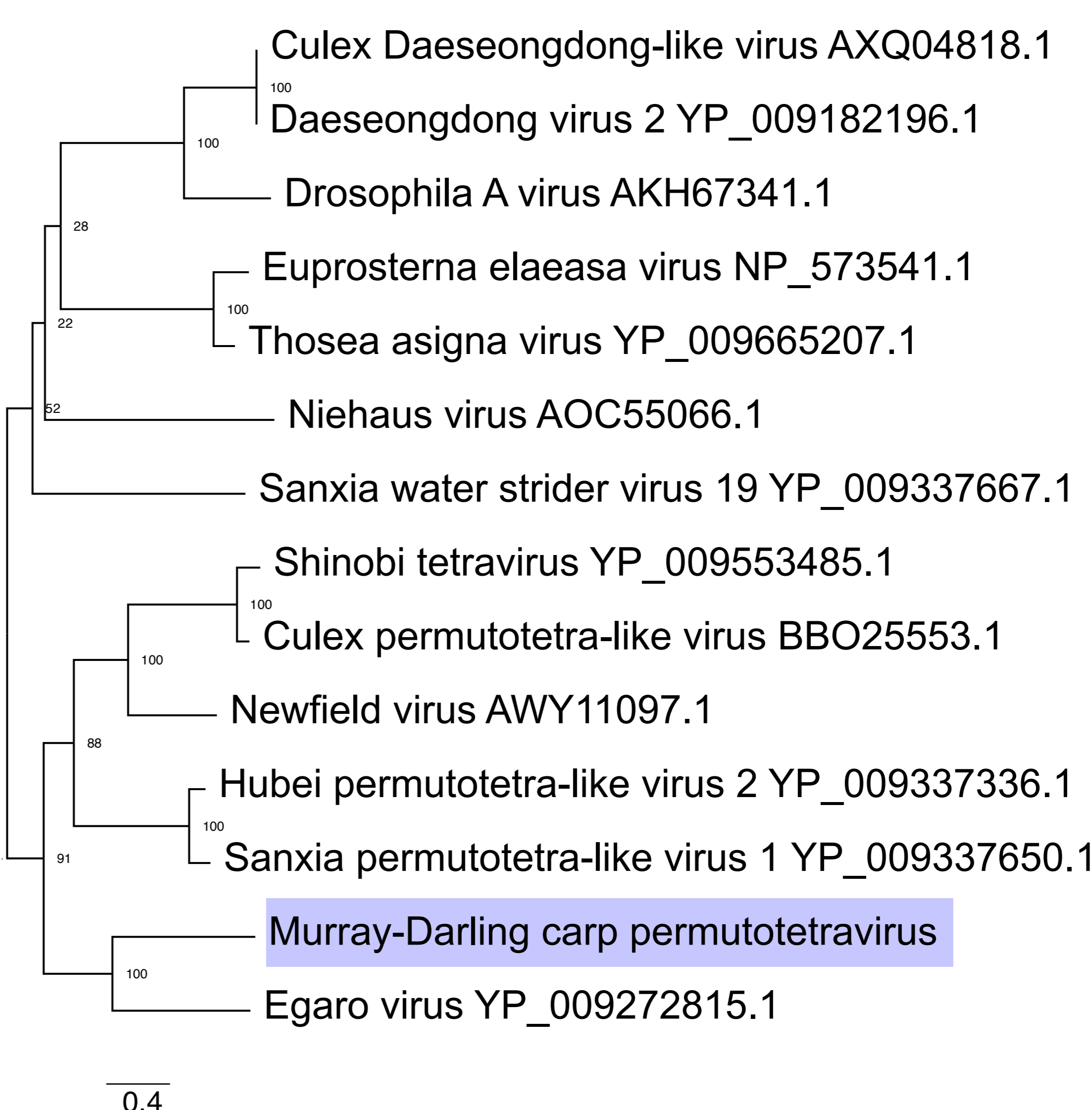

Supplement: veab034_Supplementary_Data [file veab034_supplementary_data.zip › SIFigure2.pdf]

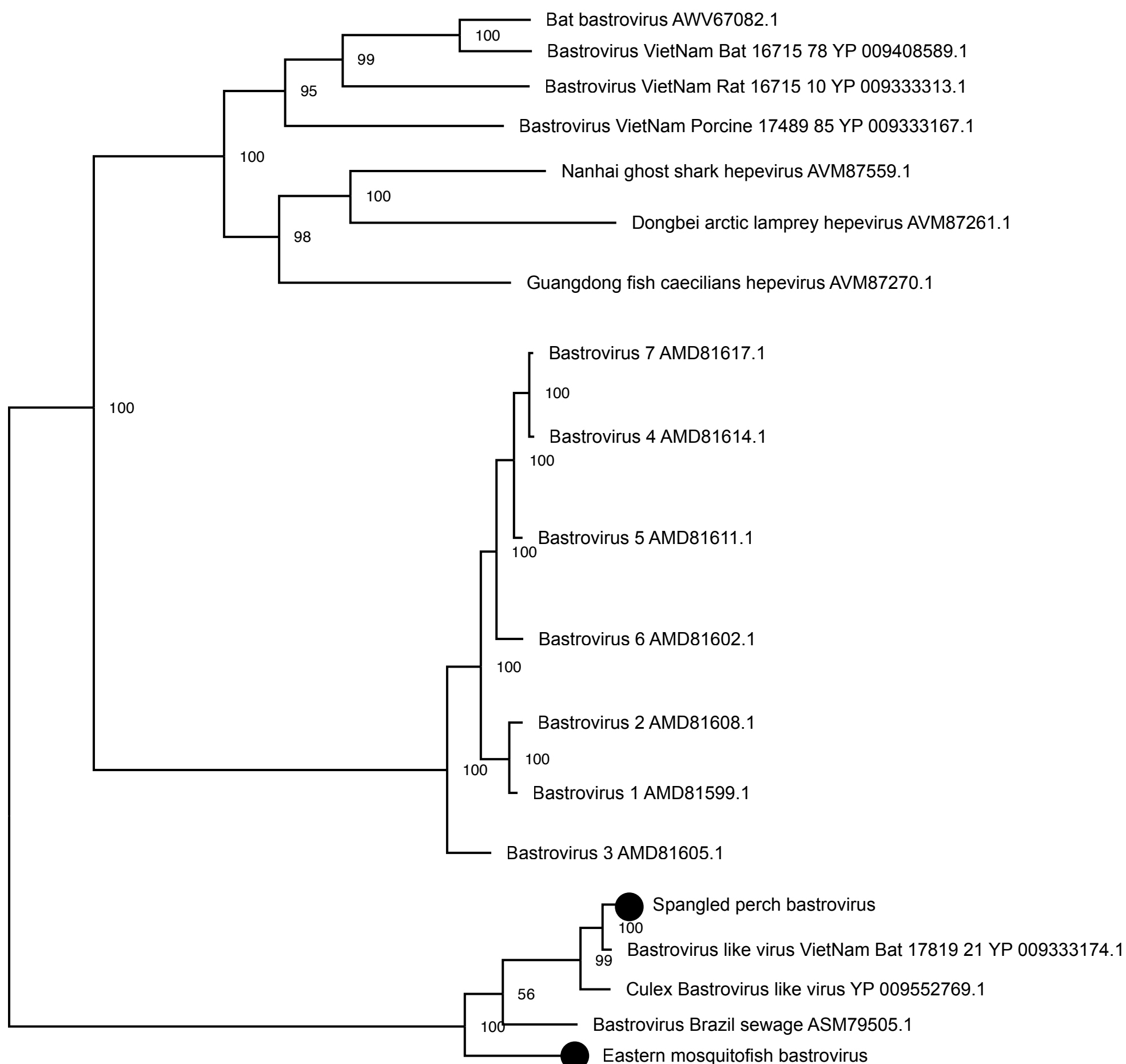

0.3

Supplement: veab034_Supplementary_Data [file veab034_supplementary_data.zip › SIFigure3.pdf]
